# Overlapping infection of *Nocardia farcinica* and *Aspergillus fumigatus* in a child with X-linked chronic granulomatous disease: a case report

**DOI:** 10.1186/s12879-021-06968-x

**Published:** 2022-01-20

**Authors:** Xiyan Tian, Qingmiao Shi, Peng Liu, Lulu Pang, Peisheng Jia, Lei Xie, Xiaoxu Ma, Ang Li, Zujiang Yu, Huaili Wang

**Affiliations:** 1 Department of Pediatric Intensive Care Unit, The First Affiliated Hospital of Zhengzhou University, Zhengzhou, China; 2 Gene Hospital of Henan Province, The First Affiliated Hospital of Zhengzhou University, Zhengzhou, China; 3 Department of Infectious Diseases, The First Affiliated Hospital of Zhengzhou University, Zhengzhou, China; 4 Department of Respiration, The First Affiliated Hospital of Zhengzhou University, Zhengzhou, China

**Keywords:** *Nocardia farcinica*, X-linked chronic granulomatous disease, Metagenomic next-generation sequencing, Whole-exome sequencing, Early diagnosis, Case report

## Abstract

**Background:**

Chronic granulomatous disease (CGD) is a rare inherited primary immunodeficiency syndrome, manifested as recurrent infections and inflammatory complications. Although prophylactic treatment with antibiotics and antifungals improved the outcome of CGD patients, infections remain the major cause of mortality.

**Case presentation:**

A boy aged 3 years and 8 months was admitted to hospital complaining of lip swelling with fever for half a month and neck abscess for 11 days. After a thorough examination, severe pneumonia, respiratory failure, oral and maxillofacial space infection, and perianal abscess were confirmed. However, his condition didn’t improve after initial comprehensive therapy. Subsequently, overlapping infections of *Nocardia farcinica* and *Aspergillus fumigatus* were identified by metagenomic next-generation sequencing. He was treated with imipenem, linezolid, and voriconazole intravenously, plus taking oral compound sulfamethoxazole. Later, his condition improved. Through whole-exome sequencing, the child was ultimately diagnosed as X-linked chronic granulomatous disease (X-CGD) caused by CYBB gene mutation. Allogeneic hematopoietic stem cell transplantation was the potential sanative approach but there were no available human leukocyte antigen compatible donors for the child. The family requested to transfer to a superior hospital for further treatment. Two months later, we followed up the child’s family. Unfortunately, the child had expired due to severe infection.

**Conclusion:**

To our knowledge, this is the first case of overlapping infection of *Nocardia farcinica* and *Aspergillus fumigatus* identified by metagenomic next-generation sequencing in a child with X-CGD from China. For infectious pathogens that are hard to diagnosis by traditional detection methods, metagenomic next-generation sequencing is recommended as an adminicle or indispensable approach for microbial identification. Patients with X-CGD have poor prognosis, early diagnosis and intervention of X-CGD may reduce the mortality.

## Background

Chronic granulomatous disease (CGD) is a rare and fatal primary phagocyte dysfunction disease [1, 2]. The incidence of CGD in the west is about 1 in 200,000 to 1 in 250,000. However, its morbidity in China has not yet been eliminated. Due to the deficiency of nicotinamide adenine dinucleotide phosphate oxidase activity, phagocytes cannot kill peroxidase-positive bacteria and fungi, leading to repeated severe infections. Up to now, the reported pathogenic genes include X-linked recessive inherited gene CYBB, which account for about 60%, as well as autosomal recessive inherited genes CYBA, NCF1, NCF2, and NCF4 [3].

CGD mostly manifests as recurrent infections and inflammatory complications [4–6]. In North America, *Aspergillus* is the most major pathogen, followed by *Staphylococcus*, *Burkholderia*, *Serratia*, and *Nocardia* [7]. However, the European research found *Staphylococcus aureus*, *Aspergillus*, and *Salmonella* were the most frequently cultured microorganisms of CGD [8]. Whereas different from that, *Aspergillus* and *Mycobacterium tuberculosis* are most common pathogens in China [9]. Here, we report a case of overlapping infection of *Nocardia farcinica* and *Aspergillus fumigatus* identified by metagenomic next-generation sequencing (mNGS) and ultimately diagnosed as X-linked chronic granulomatous disease (X-CGD) through whole-exome sequencing (WES).

## Case presentation

On July 28, 2020, a boy aged 3 years and 8 months was admitted to pediatric intensive care unit with a complaint of lip swelling with fever for half a month and neck abscess for 11 days. Physical examination revealed hyperthermia (38.7℃), tachycardia (165 bpm), tachypnea (60 bpm) and normal blood pressure (102/74 mmHg) on the day of admission. His jaw to right upper lip was swollen, with tenderness and elevated skin temperature. Palpation suggested hepatomegaly and splenomegaly. Five anal fistulas could be seen and there were ulceration and scabs of the perianal skin.

Laboratory findings revealed elevated white blood cell count of 39.79 × 10^9^/L, percentage of neutrophils 87%, procalcitonin 3.48 ng/mL, C-reactive protein 221.6 mg/L, erythrocyte sedimentation rate 44.00 mm/h, 1,3-β-D-glucan118.40 pg/mL, and galactomannan 1.06 ug/L. While the hemoglobin 95.8 g/L, percent of lymphocytes 7.7%, serum potassium 2.88 mmol/L, serum sodium 128.0 mmol/L, and albumin 23.8 g/L were reduced. Arterial blood gas analysis showed hypoxemia of 51 mmHg. Other indicators, including neutrophil oxidative burst test, T-SPOT, blood culture, bone marrow aspiration cytology, bone marrow culture, mycoplasma titer, human parvovirus, antistreptolysin “O”, and anti-neutrophil cytoplasmic antibody were negative.

**Fig. 1:**
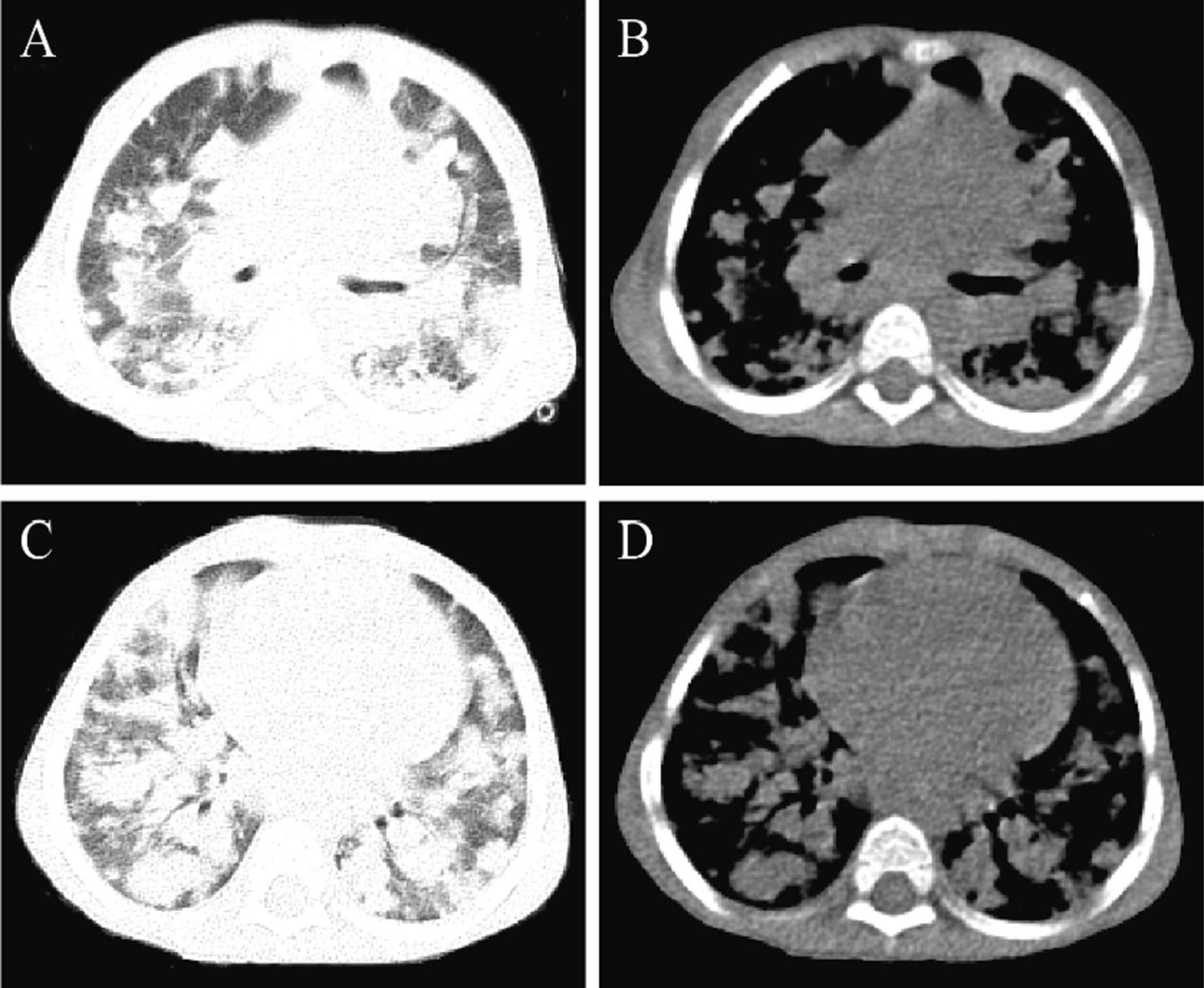
Chest CT showed multiple clusters and nodular high-density shadows in both lung fields. **A** and **C** were lung window images; **B** and **D** were corresponding mediastinum window images

Computed tomography (CT) scan indicated multiple clusters and nodular high-density shadows in both lung fields (Fig. 1), which were considered as bilateral lung infection. Besides, cystic density shadows were seen in the right maxillofacial region and submaxillary. Ultrasound found multiple enlarged lymph nodes on the bilateral neck, the larger ones were 12 mm × 6 mm on the right and 11 mm × 7 mm on the left. The right submaxillary region detected a hypoechoic area of about 35 mm × 22.5 mm, and the submental continuation to the left submaxillary region probed a range of about 79 mm × 15 mm hypoechoic.

Severe pneumonia, respiratory failure, oral and maxillofacial space infection, and perianal abscess were confirmed. The child received empirical anti-infective treatment with intravenous meropenem, vancomycin, and fluconazole. However, in the early morning of the second day of admission, the child developed breathlessness and decreased oxygen saturation. Continuous positive airway pressure (CPAP) noninvasive ventilator was applied with the informed consent of the family. The following day, he was administered nasotracheal intubation and ventilator support due to hypoxic saturation (93%) and severe carbon dioxide retention (98 mmHg).

The child’s condition didn’t improve after initial therapy and an infrequent pathogen was doubted. On day 3 post admission, submandibular pus puncture was performed and the pus was tested through metagenomic next-generation sequencing (mNGS). Within 24 h, it revealed 87 sequence readings of *Nocardia farcinica* genome (Table 1). The child was given oral compound sulfamethoxazole tablets. Besides, vancomycin was switched to linezolid and meropenem was changed to imipenem. 48 h later, special bacterial smear of pus indicated that weak acid-fast staining was positive, which was suspected of *Nocardia*. Moreover, the pus was cultured and the *N. farcinica* was identified after 5 days, further proving the pathogen detected by mNGS.

**Table 1:** The metagenomic next-generation sequencing results of the patient

Date	Specimen type	Pathogen	Sequence readings
2020.07.31	Pus	*Nocardia farcinica*	87
2020.08.03	BALF	*Nocardia farcinica*	64
		*Aspergillus fumigatus*	5

On day 5 of admission, bronchoalveolar lavage fluid (BALF) was detected by mNGS and the results showed 64 sequence readings of *N. farcinica* genome and 5 sequence readings of *Aspergillus fumigatus* genome (Table 1). While the galactomannan value reported from BALF was ≤ 0.25. The child was considered an overlapping infection of *N. farcinica* and *A. fumigatus*. Moreover, special bacterial smear of BALF found positive weak acid-fast staining, which was suspected *Nocardia*. The antibiotics were adjusted to imipenem, linezolid, and voriconazole intravenously, plus taking oral compound sulfamethoxazole. Later, the child’s condition improved.

**Fig. 2:**
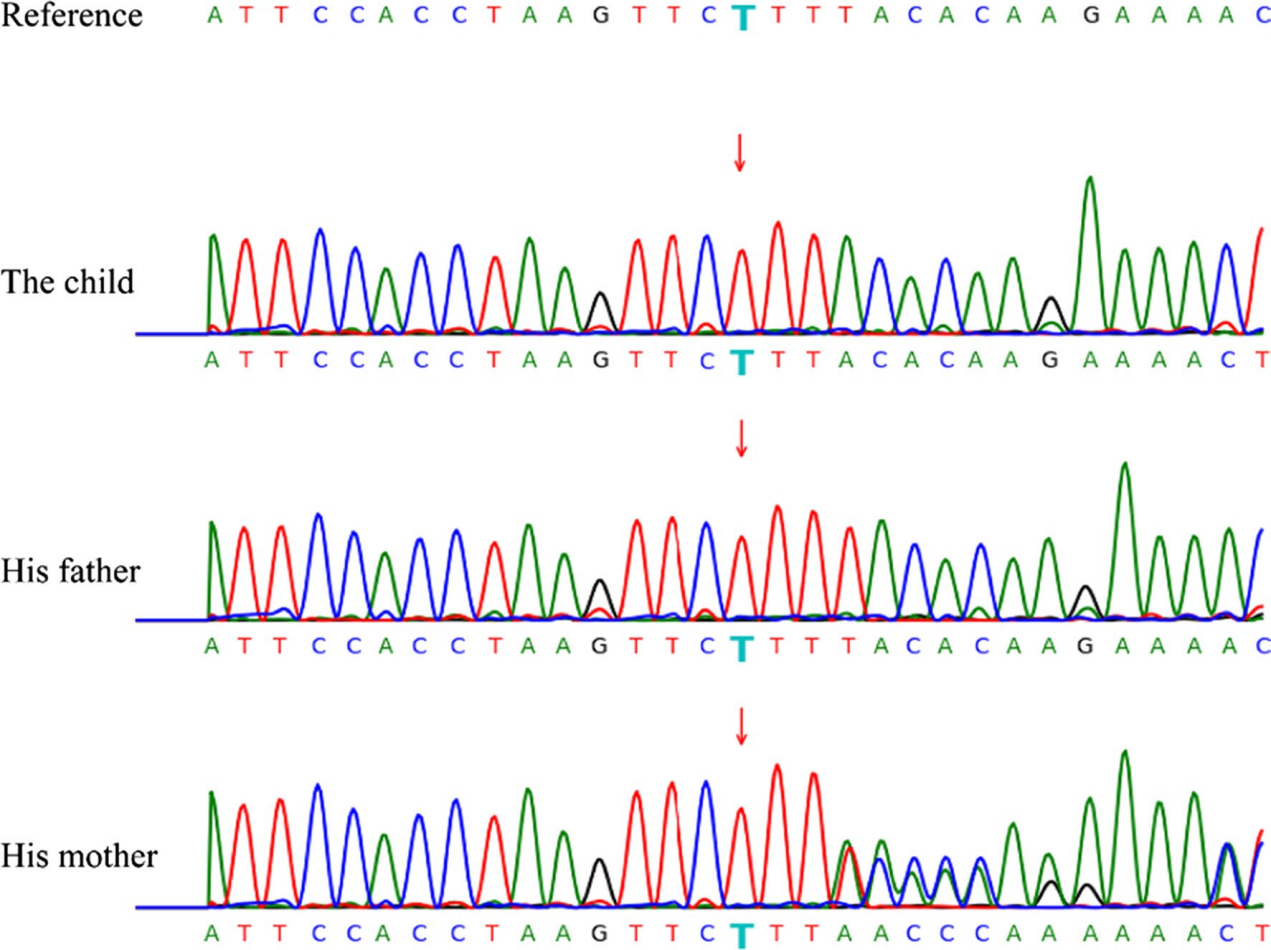
WES revealed T deletion mutation in the CYBB gene of the child and his mother

Pursuing his medical history uncovered that he developed a fever 21 days after birth and was diagnosed with pulmonary *Aspergillus* infection. Perianal abscess appeared when he was 8 months old. It’s necessary to be alert to immunodeficiency diseases. Thus, on the 8th day of admission, peripheral blood of the children and his parents were collected for whole-exome sequencing (WES). Twelve days later, WES reported c.121 locus T deletion mutation in the CYBB gene of the child (Fig. 2), which is related to X-linked chronic granulomatous disease (X-CGD). And his mother was found a carrier of X-CGD with the inherited c.121 locus T deletion mutation.

**Fig. 3:**
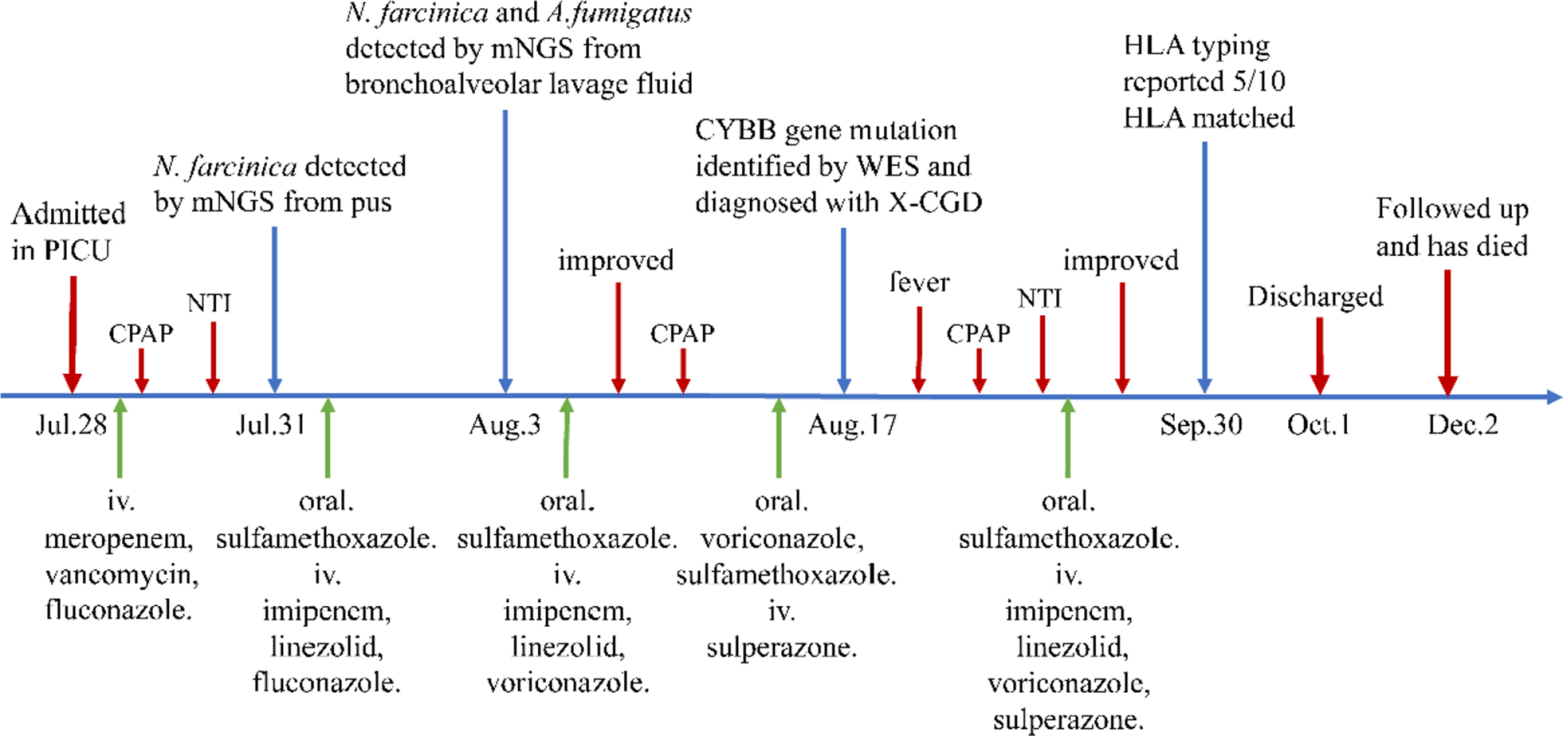
The detailed diagnosis and treatment process of the child. Abbreviations: PICU, pediatric intensive care unit; CPAP, continuous positive airway pressure; NTI, nasotracheal intubation; mNGS, metagenomic next-generation sequencing; WES, whole-exome sequencing; X-CGD, X-linked chronic granulomatous disease; HLA, human leukocyte antigen

Combined with medical history, physical signs, auxiliary examinations, pathogen detection and genetic sequencing results, the child was diagnosed as X-CGD caused by CYBB gene mutation. Allogeneic hematopoietic stem cell transplantation (HSCT) was a potential sanative approach and his family expressed willingness to have a try. On September 19, the child’s father and elder brother were administered human leukocyte antigen (HLA) typing test. On September 30, HLA typing results showed the child was 5/10 HLA-matched with his father or brother. There were no available HLA-compatible donors for the child. On October 1, the family requested to transfer to a superior hospital for further treatment. After informing them of the precautions in detail, the child was discharged. Two months later, we followed up with the child’s family. Unfortunately, the child had expired due to severe infection. The detailed diagnosis and treatment process are shown in Fig. 3.

## Discussion and conclusions

*Nocardia* species are gram-positive bacteria that grow aerobically and present various degrees of acid resistance [10]. *Nocardia farcinica*, a species of Nocardia, is a long-neglected opportunistic bacterium of high mortality rate, which is prone to missed diagnosis owing to complexity of taxonomy and inexperience in traditional identification techniques [11, 12]. In this case, mNGS was used to quickly and accurately detect that the child had both *N. farcinica* and *A. fumigatus* infection, thus providing effective assistance in diagnosis and treatment.

Accumulated evidence indicated that the mNGS has great advantages of wide coverage and higher sensitivity, which can identify multiple pathogens at one time, especially for pathogens with low positive rate of traditional testing methods [13–15]. According to a previous retrospective study, 14 samples from the patients diagnosed with nocardiosis were tested by both mNGS and culture. All 14 samples were positive for *Nocardia* by mNGS, but only 5 samples had positive culture results [16]. Another research reported a brain abscess caused by *Nocardia* diagnosed by Ziehl–Neelsen stain and mNGS, while the blood culture was negative [17]. However, the optimal timing for mNGS detection has not yet been determined, and the low specificity of mNGS also bring some challenges to clinical diagnosis [18].

In China, patients with CGD are predominantly male and generally have symptoms at an early age [9]. However, most cases were delayed diagnosed because of limited knowledge, leading to relative high mortality. The child in our case was infected with *Aspergillus* at 21 days of life and occurred perianal abscess at 8 months old, which were earliest clues of CGD. Whereas, this X-linked recessive hereditary disease was not identified until this admission, reminding us that our understanding of CGD needs to be improved. In this case, whole-exome sequencing acted an important function in the diagnosis of inherited diseases. Once CGD is suspected, whole-exome sequencing is a valid way to clarify the diagnosis.

Although prophylactic treatment with antibiotics and antifungals improved outcomes of CGD patients, infections remain a major cause of mortality. Particularly, previous study found that X-CGD patients generally had poorer survival than autosomal recessive CGD [8]. Prompt and effective therapy is of great importance. HSCT proved to be a practicable method to rescue CGD patients [19]. However, HSCT has its own limitations, such as limited donor availability, risks of pretreatment radiotherapy and chemotherapy, and graft-versus-host disease. Lentiviral gene therapy for X-CGD is in research but has not been widely implemented [20]. Our pediatric patient was haploidentical with his father or brother, especially because he had severe infections, causing high risks of conducting HSCT. Unfortunately, the child eventually died of multiple infectious complications and lost the opportunity for further treatment.

This case has some limitations. In the multidisciplinary diagnosis and treatment discussion, it was raised that the selection of empirical treatment was improper. The child was initially given antifungal treatment with fluconazole after admission. Fluconazole is a hydrophilic drug with small molecular size and low protein binding ratio, so it is the first choice for urinary tract and abdominal *Candida* infection. However, the child was presented with lung infection and he had a history of *Aspergillus* infection. Thus, *Aspergillus* should be considered in the initial antifungal therapy. After the mNGS indicated *A. fumigatus* infection, the fluconazole was changed to voriconazole. Voriconazole is a lipophilic drug with a wide distribution in lung tissue, alveoli, and epithelial lining fluid, making it the drug of choice for pulmonary *Aspergillus*.

To our knowledge, this is the first case of an overlapping infection of *Nocardia farcinica* and *Aspergillus fumigatus* identified by mNGS in an X-CGD child from China. For infectious pathogens hard to diagnosis by traditional detection methods, mNGS is recommended as an adminicle or indispensable approach for identifying microorganisms. The patient with X-CGD has a poor prognosis, early diagnosis and intervention of X-CGD may reduce mortality.

## Data Availability

All the data and materials in this report are from the authors on reasonable request.

## Abbreviations

CGD: Chronic granulomatous disease
X-CGD: X-linked chronic granulomatous disease
mNGS: Metagenomic next-generation sequencing
WES: Whole-exome sequencing
HSCT: Hematopoietic stem cell transplantation
HLA: Human leukocyte antigen
CPAP: Continuous positive airway pressure
